# XDR Tuberculosis: A Looming Threat

**DOI:** 10.4103/0970-0218.51215

**Published:** 2009-04

**Authors:** Vishal Sharma, Sourabh Aggarwal

**Affiliations:** Department of Medicine, University College of Medical Sciences, Delhi, India. E-mail: docvishalsharma@gmail.com

Sir,

Grover, *et al.* have reviewed the recent advances in drug resistant tuberculosis.(1) In this regard, it is important not to forget the recent threat of extensively drug resistant (XDR) tuberculosis that is resistant to isoniazid, rifampin, a fluoroquinolone, and any injectable drug (aminoglycosides or polypetides).(2) The cases of XDR have been reported from across the globe. It is also a cause of major concern in India.(3) This strain requires multiple second-line drugs for treatment and early reports indicated very high case fatality rates. This was probably due to concurrent HIV infection.(2) In this entire gloomy scenario, there is at least one silver lining from the recent report from Peru. In that report, individualized treatment of HIV-negative XDR cases yielded results comparable to MDR tuberculosis.(4) The importance for India lies in the fact that Peru, much like us, is a developing country.

The objective for us, however, is to prevent the emergence and transmission of such a strain. The most important risk factor implicated in the occurrence of XDR cases is inadequate or inappropriate management of tuberculosis. Hence, there is an urgent need to strengthen services of DOTS and DOTS-Plus under RNTCP. Only an efficient first-line therapy in the form of DOTS can stop XDR tuberculosis from being a major public health concern in the days to come.
